# How do health literacy and chronic disease influence the diagnostic evaluation of patients with lung cancer symptoms?

**DOI:** 10.2340/1651-226X.2025.44113

**Published:** 2025-10-23

**Authors:** Lisa Maria Sele Sætre, Kirubakaran Balasubramaniam, Sonja Wehberg, Christian Borbjerg Laursen, Jens Søndergaard, Dorte Ejg Jarbøl

**Affiliations:** a Research Unit of General Practice, Department of Public Health, University of Southern Denmark, Odense, Denmark; b Odense Respiratory Research Unit, Odense University Hospital, Department of Clinical Research, University of Southern Denmark, Odense, Denmark; c Department of Respiratory Medicine, Odense University Hospital, Odense, Denmark

**Keywords:** Lung neoplasms, diagnosis, general practice, diagnostic imaging, social inequity

## Abstract

**Background and purpose:**

Smoking status, health literacy challenges and chronic diseases may influence the diagnostic evaluation of patients presenting with lung cancer symptoms (LCSs) in general practice. This study aimed to (1) analyse associations between smoking status, health literacy, chronic disease and having completed diagnostic imaging amongst patients with LCSs in Danish general practice and (2) investigate how these factors interact in relation to the completion of diagnostic imaging, by examining effect modification and causal mediation.

**Patient/material and methods:**

In 2022, a random sample of 100,000 individuals aged ≥ 20 years from the Danish population was invited to participate in a survey about symptoms and healthcare seeking. This study included individuals aged ≥ 40 years who reported general practitioner (GP) contact with LCSs. Questionnaire data included health literacy, chronic disease and smoking status. Register data included socioeconomics, prescription drugs and diagnostic imaging. Descriptive statistics, multivariable logistic regression and causal mediation models were applied.

**Results:**

Of the 2,252 patients who had contacted their GP with LCSs, 22% had completed diagnostic imaging. Formerly smoking increased odds of diagnostic imaging compared to never smoking, whereas current smoking had no influence. No associations or mediations were demonstrated between health literacy, chronic disease and diagnostic imaging. Effect modification was implied by varying impact of health literacy on diagnostic imaging depending on smoking status, yet the results were limited by power.

**Interpretation:**

Initiatives targeting awareness of the risk of overlooking cancer symptoms amongst high-risk patients presenting in general practice may improve the chance of timely diagnosis of lung cancer.

## Introduction

Despite developments in treatment options and improved survival rates, lung cancer remains the leading cause of cancer-related death worldwide [1]. Screening has been implemented in some countries, but most lung cancer diagnostics must still be symptom based [2]. Recommendations for when general practitioners (GPs) should refer patients with lung cancer symptoms (LCSs) for diagnostic imaging are included in national and international cancer care pathways (CCPs) [3, 4]. Most existing evidence on diagnostic evaluation is based on register data, patients diagnosed with lung cancer and anticipated referral patterns [5, 6]. Consequently, the evidence does not account for the patients who contact general practice with LCSs but are not later diagnosed with lung cancer.

Despite free and universal provision of health services in Denmark, inequity in lung cancer persists [7]. Approximately 80% of all lung cancers are caused by the use of tobacco [8, 9], and smoking is related to 80–90% of inequities in cancer [9]. Due to a higher a priori risk of cancer, patients with a smoking history are expected to be more likely to undergo diagnostic evaluation for lung cancer than patients without a smoking history when presenting LCSs in general practice. However, other factors may modify or mediate the impact of smoking history on diagnostics [6, 10].

Health literacy has been suggested as a dynamic outcome of socioeconomic factors contributing to health inequities [11]. It is defined as the combination of the personal competencies and situational resources needed for people to access, understand, appraise and use information and services to make decisions about health, including the capacity to communicate, assert and act upon these decisions [12]. Health literacy can be evaluated using different methodologies. In this study, we used the Health Literacy Questionnaire (HLQ) [13]. Existing chronic disease, and perhaps particularly respiratory diseases, may influence the interpretation of symptoms and the decision of diagnostic evaluation [14, 15]. Health literacy challenges as well as chronic disease have been associated with current smoking [16, 17], less healthcare seeking [18] and cancer outcomes such as prolonged time from symptom recognition to diagnosis, lower screening participation, non-adherence to treatment and higher mortality [19].

In this study, we hypothesised that health literacy challenges and chronic disease reduce the probability of completing diagnostic imaging for patients presenting with LCSs in general practice. Moreover, we hypothesised that health literacy and chronic disease may mediate the likelihood of individuals with different smoking history completing diagnostic evaluation. Therefore, this population-based study aimed to (1) analyse associations between smoking status, health literacy, chronic disease and having completed diagnostic imaging amongst patients with LCSs in Danish general practice and (2) investigate how these factors interact in relation to the completion of diagnostic imaging, by examining effect modification and causal mediation.

## Patients/material and methods

### Study sampling and logistics

This study is based on the Danish Symptom Cohort II (DaSC II) study. A total of 100,000 individuals aged ≥ 20 years were randomly selected through the Danish Civil Registration System (CRS) and invited to participate in a survey about symptoms and healthcare-seeking behaviour. In the CRS, all individuals living in Denmark are provided with a unique identification number (CRS number), which is assigned to all Danish citizens upon birth or immigration. Invitations were sent to a digital mailbox linked to the CRS number. Reminders were sent to non-respondents after 7 and 14 days. Data were collected from May to July 2022. The study sampling and logistics have been described in detail elsewhere [20].

### Setting

More than 98% of all Danish citizens are listed with a general practice. GPs serve as gatekeepers to both out- and inpatient hospital contacts, private practising specialists and other healthcare providers, including CCPs [21]. The Danish lung CCP includes specific and non-specific symptoms, which should lead to suspicion of lung cancer amongst individuals who are 40 years or older with a relevant history of tobacco use and prompt the GP to refer the patient for diagnostic imaging in terms of computed tomography of the thorax (CT thorax) [4]. Nevertheless, chest X-ray (CXR) is also commonly used as the first choice of imaging.

### Questionnaire development and data

The development of the DaSC II questionnaire followed the COnsensus-based Standards for the selection of health Measurement Instruments [22]. The questionnaire was pilot tested twice and field tested amongst 499 randomly selected individuals from the general population. Overall, the questionnaire showed good comprehensibility and content validity. The conceptual framework and development have been described elsewhere [20].

In the present study, we included data on contact to general practice with five specific LCSs (prolonged coughing [> 4 weeks], dyspnoea, haemoptysis, prolonged hoarseness [> 4 weeks], changes in a familiar cough) experienced within the 4 weeks preceding data collection [4].

Health literacy was assessed using four domains from the HLQ [13], which has been translated into Danish, culturally adapted and validated [23]. The four domains were selected based on the hypothesis that they exert the greatest influence within a primary healthcare setting, to keep the length of the DaSC II questionnaire manageable [16] and the domains reflected the following aspects of health literacy: feeling understood and supported by healthcare providers (‘Understood and supported’, 5 items); having sufficient information to manage my health (‘Sufficient information’, 4 items); having social support for health (‘Social support’, 5 items); and the ability to actively engage with healthcare providers (‘Actively engage’, 5 items) [16]. The domains encompass 18 items covering a range of statements. For the first three domains, the respondents were asked on a four-point Likert scale whether they agreed with each statement (1 = strongly disagree, 2 = disagree, 3 = agree, 4 = strongly agree), and for the last domain, responses were given on a five-point Likert scale from difficult to easy (1 = always difficult, 2 = usually difficult, 3 = sometimes difficult, 4 = usually easy, 5 = always easy) [13].

Having any existing chronic disease was self-reported. The respondents were asked whether they had a pre-existing disease, injury or disability. Individuals who answered ‘don’t know’ were interpreted as having no chronic disease. Smoking status was categorised as never, former and current smoking [24].

Details on the questionnaire data are described in the Supplementary material, Table S1.

### Register data

The survey data were linked to register data using the CRS number. Data on sex, age and vital status were obtained from the Danish Health Data Authority. Data on marital status, highest obtained level of education, labour market affiliation and ethnicity were obtained from Statistics Denmark.

To evaluate the presence of chronic respiratory diseases, we utilised data from the Register of Pharmaceutical Sales on prescriptions for medications commonly used in the treatment of asthma and Chronic Obstructive Pulmonary Disease. Patients who had redeemed at least two prescriptions for a relevant drug within the year preceding data collection were classified as having a chronic respiratory disease [25]. The drugs considered included bronchodilators, corticosteroids and leukotriene modifiers.

Data on diagnostic evaluations were obtained from the Danish National Patient Register and included diagnostic imaging in terms of CXR (Codes: UXRC and UXRC00) and CT thorax (Codes: UXCC, UXCC00 and UXCC75).

Details on registers and codings are provided in the Supplementary material, Table S1.

### Study population

Individuals who died, emigrated or were exempted from digital mail were considered ineligible. To comply with the Danish lung cancer guideline, individuals < 40 years old were excluded. We applied listwise deletion to missing questionnaire data, whereas missing data on socioeconomic status (< 0.1%) were recoded to the largest group within each variable. Only respondents who had presented at least one LCS to their GP were included in the analyses.

### Outcome

**Figure 1:**
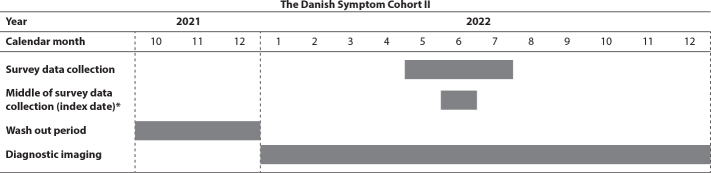
Timeline for data collection.

Outcome was completion of at least one diagnostic imaging, in terms of either a CXR or a CT thorax. Diagnostic evaluation was assessed 6 months prior to and post-survey data collections in the period from 1^st^ January 2022 to 31^st^ December 2022, Figure 1. To ensure that the imaging conducted within this period was the first in the specific diagnostic course, all patients who had completed diagnostic imaging during a wash out period comprising the 3 months prior to the period of interest (1^st^ October 2021 to 31^st^ December 2021) were coded as not having completed diagnostic imaging, Figure 1. Procedures that had been performed acutely were not included in the outcome, as they are linked to visits to the emergency department [26]. For individuals who had completed more than one imaging, only the first was used.

*Index date: June 1st, 2022.

### Covariates

The covariates included the following explanatory variables: sex: male or female; age groups: 40–54, 55–69 or 70 years or older; smoking status: never, former or current; chronic disease: yes or no; chronic respiratory disease: yes and no; health literacy; potential confounders including symptom burden: 1 symptom, 2–3 symptoms or ≥ 3 symptoms; marital status: single/living alone or married/cohabiting; highest obtained level of education: low (< 10 years), middle (10–15 years) or high (≥ 15 years); labour market affiliation: working or out of workforce; and ethnicity: Danish or immigrants/descendants of immigrants.

### Statistical analyses

Study population characteristics were calculated by using descriptive statistics. Mean score and standard deviations (SDs) for each of the four HLQ domains were calculated as the sum score within each domain divided by the number of items.

Multivariable logistic regression models were applied to analyse the associations between the explanatory variables and diagnostic imaging. Adjustments were made in two steps. First, in model A, we adjusted for sex, age, smoking status, symptom burden, self-reported chronic disease and chronic respiratory disease. Second, in model B, we additionally adjusted for socioeconomic factors. The models were evaluated with C-statistics estimating Area Under the Curve (AUC) and 95% confidence intervals (CIs).

Effect modification was assessed by repeating the multivariable logistic regression models stratified on smoking status. Whether health literacy or chronic disease acted as a mediator of the effect of smoking status was evaluated in causal mediation models using the mediate function in Stata version 18. We estimated six causal mediation models, with each of the four health literacy aspects (continuous variable), chronic disease (dichotomous variable) and chronic respiratory disease (dichotomous variable) respecitvely, as mediators, completed imaging as outcome (dichotomous variable) and smoking status as explanatory variable (two dichotomous variables: never vs. former and never vs. current), and adjusted for potential confounders, Supplementary Figure S1. The model decomposed the total effect into a direct and indirect effect, expressed as odds ratios (ORs).

Data analyses were conducted using Stata version 18 (StataCorp, USA). All tests used a significance level of *p* < 0.05.

Reporting followed the STROBE checklist (Supplementary material, Table S2).

## Results

**Figure 2:**
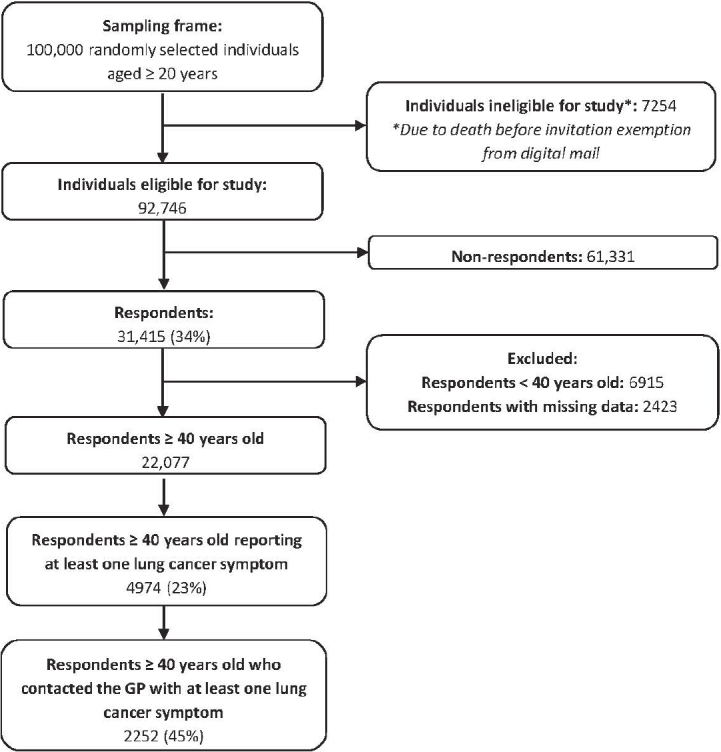
Flowchart of the study population.

Of the 100,000 randomly selected individuals, 7,254 were ineligible for study and 31,415 (34%) responded to the questionnaire. Amongst these, 22,077 were ≥40 years and had completed questionnaire data. A total of 4,974 (22.5%) individuals reported at least one LCS, and of these, 2,252 (45.3%) had contacted their GP, Figure 2. Characteristics of the study population are shown in Table 1.

**Table 1:** Characteristics of the respondents and study population.

	Respondents≥ 40 years old	Respondents reporting at least one lung cancer symptom	Respondents reporting GP contact with at least one lung cancer symptom
	*n* (%)	*n* (%)	*n* (%)
**Total**	22,077 (100.0)	4,974 (100.0)	2,252 (100.0)
**Sex**			
Female	12,220 (55.4)	2,685 (54.0)	1,284 (57.0)
Male	9,857 (44.6)	2,289 (46.0)	968 (43.0)
**Age groups**			
40–54 years	6,884 (31.2)	1,317 (26.5)	474 (21.0)
55–69 years	9,386 (42.5)	2,217 (44.6)	994 (44.1)
70+ years	5,807 (26.3)	1,440 (29.0)	784 (34.8)
**Smoking status**			
Never smoking	6,884 (31.2)	1,317 (26.5)	875 (38.9)
Former smoking	9,386 (42.5)	2,217 (44.6)	1,020 (45.3)
Current smoking	5,807 (26.3)	1,440 (29.0)	357 (15.9)
**Self-reported chronic disease**			
No	11,949 (54.1)	2,045 (41.1)	745 (33.1)
Yes	10,128 (45.9)	2,929 (58.9)	1,507 (66.9)
**Chronic respiratory disease**			
No	20,249 (91.7)	3,907 (78.5)	1,499 (66.6)
Yes	1,828 (8.3)	1,067 (21.5)	753 (33.4)
**Health literacy**	**Mean score (SD)**	**Mean score (SD)**	**Mean score (SD)**
Understood and supported	2.90 (0.67)	2.85 (0.68)	2.90 (0.67)
Sufficient information	3.02 (0.56)	2.90 (0.57)	2.89 (0.60)
Social support	3.04 (0.59)	2.94 (0.59)	2.96 (0.60)
Actively engage	3.80 (0.87)	3.61 (0.92)	3.61 (0.93)
**Symptom burden**			
0 symptoms	17,103 (77.5)	-	-
1 symptom	3,648 (16.5)	3,648 (73.3)	1,441 (64.0)
2 symptoms	1,093 (5.0)	1,093 (22.0)	643 (28.6)
≥ 3 symptoms	233 (1.1)	233 (4.7)	168 (7.5)
**Marital status**			
Single	5,923 (26.8)	1,526 (30.7)	714 (31.7)
Married/cohabiting	16,154 (73.2)	3,448 (69.3)	1,538 (59.3)
**Highest obtained educational level**			
Low (< 10 years)	5,923 (26.8)	1,526 (30.7)	714 (31.7)
Middle (10–15 years)	16,154 (73.2)	3,448 (69.3)	1,538 (68.3)
High (> 15 years)	5,923 (26.8)	1,526 (30.7)	714 (31.7)
**Labour market affiliation**			
Working	12,592 (57.0)	2,487 (50.0)	952 (42.3)
Out of workforce	9,485 (43.0)	2,487 (50.0)	1,300 (57.7)
**Ethnicity**			
Danish	20,796 (94.2)	4,684 (94.2)	2,115 (93.9)
Immigrants or descendants of immigrants	1,281 (5.8)	290 (5.8)	137 (6.1)

GP: general practitioner; SD: standard deviation.

Table 2 shows the proportion of patients who had completed diagnostic imaging and associations with each explanatory variable. A total of 490 (21.8%) patients had completed diagnostic imaging. Proportions were highest amongst patients who formerly smoked (248, 24.3%) and in the oldest age group (204, 26.0%).

**Table 2:** Proportion of patients who completed diagnostic evaluation amongst patients presenting with lung cancer symptoms in general practice and logistic regression estimates, odds ratios (OR) and corresponding 95% confidence intervals (CI) for both crude and adjusted models (*N* = 2,252).

	*n* (%)	Crude OR(95% CI)	Model A*:Adjusted OR(95% CI)	Model B*:Adjusted OR(95% CI)
**Total imaging**	490 (21.8)	-	-	-
**Sex**				
Female	273 (21.7)	1	1	1
Male	217 (22.4)	1.07 (0.87–1.31)	1.00 (0.81–1.23)	0.99 (0.80–1.23)
**Age groups**				
40–54 years	73 (15.4)	1	1	1
55–69 years	213 (21.4)	1.50 (1.12–2.01)	1.46 (1.09–1.96)	1.33 (0.98–1.81)
70+ years	204 (26.0)	1.93 (1.44–2.60)	1.88 (1.38–2.54)	1.57 (1.09–2.25)
**Smoking status**				
Never smoking	166 (19.0)	1	1	1
Former smoking	248 (24.3)	1.37 (1.10–1.71)	1.30 (1.03–1.63)	1.28 (1.02–1.60)
Current smoking	76 (21.3)	1.16 (0.85–1.57)	1.16 (0.85–1.58)	1.11 (0.81–1.53)
**Self-reported chronic disease**				
No	159 (21.3)	1	1	1
Yes	331 (22.0)	1.04 (0.84–1.28)	0.94 (0.75–1.17)	0.92 (0.74–1.15)
**Chronic respiratory disease**				
No	314 (21.0)	1	1	1
Yes	176 (23.4)	1.15 (0.93–1.42)	1.02 (0.82–1.28)	1.00 (0.80–1.26)
**Health literacy**	**Mean score (SD)**			
Understood and supported**	2.93 (0.67)	1.09 (0.94–1.27)	1.07 (0.91–1.24)	1.07 (0.92–1.25)
Sufficient information**	2.88 (0.58)	0.99 (0.84–1.17)	0.98 (0.82–1.16)	1.00 (0.84–1.19)
Social support**	2.99 (0.60)	1.09 (0.92–1.29)	1.10 (0.93–1.31)	1.12 (0.94–1.33)
Actively engage**	3.66 (0.90)	1.08 (0.96–1.20)	1.06 (0.95–1.19)	1.08 (0.96–1.20)

OR: odds ratio; CI: confidence interval.

Model A: Adjusted for sex, age, smoking status, symptom burden, self-reported chronic disease, chronic respiratory disease and health literacy*.

Model B: Adjusted for sex, age, smoking status, symptom burden, self-reported chronic disease, chronic respiratory disease and health literacy*, marital status, educational level, labour market affiliation and ethnicity.

* The regression models for the four health literacy aspects were not adjusted for the remaining aspects due to intercorrelation.

** OR per one unit increase in domain score for the health literacy aspect.

Former smoking was associated with higher odds of having completed imaging (OR 1.28, 95% CI 1.02–1.60), whereas the odds for patients who currently smoked were similar to patients who never smoked, Table 2. No associations were found between health literacy, chronic diseases and having completed diagnostic imaging, Table 2. The estimates in models A (AUC 0.59, 95% CI: 0.56–0.61) and B (AUC 0.60, 95% CI: 0.57–0.63) were similar, and AUC for both models is higher than for the crude model (AUC 0.51, 95% CI: 0.48–0.54).

Table 3 shows the analyses of the association analyses stratified on smoking status. Half of those who completed diagnostic imaging had a former smoking history (248, 50.6%), whereas only 15.5% (*n* = 76) had a current smoking history. Amongst patients who currently smoked, self-reported chronic disease reduced the odds diagnostic imaging (OR 0.53, 95% CI 0.29–0.97) compared to patients without a chronic disease who currently smoked. For patients with a chronic respiratory disease, the tendency was opposite (OR 1.34, 95% CI 0.74–2.41).

For patients who either never or currently smoked, a higher health literacy score seemed to increase the odds of having completed diagnostic imaging, although not statistically significant, except regarding the ability to actively engage with healthcare providers amongst patients without a smoking history (OR 1.25, 95% 1.03–1.52). For patients who formerly smoked, the tendencies were the opposite, Table 3.

**Table 3:** Proportion of patients who completed diagnostic evaluation after presenting lung cancer symptoms in general practice stratified on smoking status and adjusted logistic regression estimates, odds ratios (OR) with corresponding 95% confidence intervals (CI).

	Total imaging	Never smoking		Former smoking		Current smoking	
	n	*n* (%)	Adjusted OR*(95% CI)	*n* (%)	Adjusted OR*(95% CI)	*n* (%)	Adjusted OR* (95% CI)
**Total**	490	166 (33.9)		248 (50.6)		76 (15.5)	
**Sex**							
Female	273	114 (23.3)	1	119 (24.3)	1	40 (8.2)	1
Male	217	52 (10.6)	0.80 (0.55–1.16)	129 (26.3)	1.05 (0.77–1.43)	36 (7.3)	1.35 (0.79–2.31)
**Age groups**							
40–54 years	73	32 (6.5)	1	26 (5.3)	1	15 (3.1)	1
55–69 years	213	71 (14.5)	1.21 (0.75–1.96)	101 (20.6)	1.52 (0.93–2.50)	41 (8.4)	1.31 (0.65–2.64)
70+ years	204	63 (12.9)	1.25 (0.68–2.32)	121 (24.7)	2.02 (1.15–3.55)	20 (4.1)	1.44 (0.61–3.40)
**Self-reported chronic disease**							
No	159	55 (11.2)	1	75 (15.3)	1	29 (5.9)	1
Yes	331	111 (22.7)	1.11 (0.76–1.62)	173 (35.3)	0.97 (0.70–1.35)	47 (9.6)	0.53 (0.29–0.97)
**Chronic respiratory disease**							
No	314	131 (41.7)	1	143 (45.4)	1	40 (12.7)	
Yes	176	35 (19.9)	0.67 (0.44–1.02)	105 (59.7)	1.13 (0.83–1.55)	36 (20.5)	1.34 (0.74–2.41)
**Health literacy**	**Mean score**	**Mean score (SD)**		**Mean score (SD)**		**Mean score (SD)**	
Understood and supported**	2.93	3.00 (0.65)	1.27 (0.98–1.64)	2.90 (0.66)	0.91 (0.73–1.15)	2.88 (0.70)	1.16 (0.78–1.73)
Sufficient information**	2.88	2.98 (0.60)	1.21 (0.90–1.61)	2.84 (0.54)	0.80 (0.62–1.05)	2.82 (0.65)	1.24 (0.80–1.94)
Social support**	2.99	3.08 (0.63)	1.28 (0.95–1.71)	2.96 (0.56)	1.01 (0.77–1.31)	2.87 (0.64)	1.08 (0.70–1.69)
Actively engage**	3.66	3.77 (0.88)	1.25 (1.03–1.52)	3.60 (0.88)	0.95 (0.80–1.11)	3.63 (0.99)	1.23 (0.93–1.62)

* Adjusted for sex, age, smoking status, symptom burden, chronic disease, chronic respiratory disease and health literacy*, marital status, educational level, labour market affiliation and ethnicity.

The regression models for the four health literacy aspects were not adjusted for the remaining aspects due to intercorrelation.

** OR per one unit increase in domain score for the health literacy aspect.

Table 4 presents the results of the causal mediation models. The indirect effect was negligible in all the models; thus, the natural direct effects and total effects were alike.

**Table 4:** The natural indirect effect (NIE) with health literacy, chronic disease and respiratory chronic disease as mediators, natural direct effect (NDE) of smoking status and total effect (TE) of smoking status on the odds of having completed diagnostic imaging amongst patients who had presented lung cancer symptoms in general practice (N = 2,252).

			Former smoking vs. never smoking	Current smoking vs. never smoking
			OR* (95% CI)	OR* (95% CI)
**Health literacy**	Understood and supported**	**NIE**	1.00 (1.00–1.01)	1.00 (0.98–1.01)
		**NDE**	1.27 (1.02–1.59)	1.13 (0.83–1.53)
		**TE**	1.28 (1.02–1.59)	1.12 (0.82–1.53)
	Sufficient information**	**NIE**	0.99 (1.00–1.00)	1.00 (0.98–1.03)
		**NDE**	1.27 (1.02–1.59)	1.13 (0.83–1.53)
		**TE**	1.27 (1.02–1.59)	1.13 (0.83–1.53)
	Social support**	**NIE**	1.00 (0.99–1.01)	0.99 (0.96–1.01)
		**NDE**	1.28 (1.02–1.59)	1.14 (0.84–1.54)
		**TE**	1.27 (1.02–1.59)	1.12 (0.82–1.52)
	Actively engage**	**NIE**	1.00 (0.99–1.01)	0.99 (0.96–1.01)
		**NDE**	1.27 (1.02–1.59)	1.13 (0.83–1.53)
		**TE**	1.27 (1.02–1.59)	1.12 (0.82–1.52)
**Chronic disease**		**NIE**	1.00 (0.98–1.01)	1.00 (0.99–1.01)
		**NDE**	1.27 (1.02–1.58)	1.12 (0.82–1.52)
		**TE**	1.27 (1.02–1.58)	1.12 (0.83–1.53)
**Chronic respiratory disease**		**NIE**	1.00 (0.97–1.03)	1.00 (0.97–1.03)
		**NDE**	1.27 (1.02–1.58)	1.12 (0.82–1.52)
		**TE**	1.27 (1.02–1.58)	1.12 (0.82–1.52)

* Adjusted for sex, age, symptom burden, chronic disease, chronic respiratory disease, health literacy, marital status, educational level, labour market affiliation and ethnicity.

The regression models for the four health literacy aspects were not adjusted for the remaining aspects due to intercorrelation.

** OR per one unit increase in domain score for the health literacy aspect.

## Discussion and conclusion

### Main findings

Approximately one out of five (22%) of the patients who had presented LCSs in general practice had completed diagnostic imaging. The proportion of diagnostic imaging was higher amongst patients with older age and those who formerly smoked. Individuals who currently smoked had similar likelihood of completing diagnostic imaging, whilst having formerly smoked increased the odds of diagnostic imaging compared to never smoking. This study did not demonstrate associations between any aspects of health literacy, existing chronic disease and completion of diagnostic imaging. Some effect modification was implied as the associations between health literacy and diagnostic imaging appear to vary by smoking status. However, the interactions lacked statistical significance. The relation between smoking history and diagnostic imaging was not mediated by any of the health literacy aspects or chronic disease.

### Discussion of results and comparison with other literature

Overall, the present study could not demonstrate disparities in diagnostic imaging with regard to health literacy and chronic disease. However, it highlights that patients who currently smoke, a group considered at high risk, are not more likely to complete diagnostic imaging. This contrasts with our hypotheses and is notable in light of the social inequities in the risk, incidence and mortality of lung cancer [9]. The results could imply that GPs evaluate their patients equally, regardless of health literacy and chronic disease. Nevertheless, differentiation of patients is necessary in relation to varying a priori risks of having a serious disease and the appropriate use of health resources. The results could also indicate that the decision of referral does not rely on the patients’ health literacy. This is supported by Banks et al., who found low patient involvement in the decision of referral for diagnostic evaluation of lung cancer in general practice [27], and by low awareness of health literacy in primary care [28]. Notably, being able to actively engage with healthcare providers tended to increase the odds of diagnostic imaging amongst patients without a former smoking history, perhaps due to higher capability of advocating for being examined.

The stratified analyses implied that patients who currently smoked and had a chronic disease were less likely to undergo diagnostic imaging, whereas the opposite was the case for patients with a chronic respiratory disease. However, these findings may be inflicted by type I error and should be interpreted with caution. The findings partly contrast with studies, suggesting that chronic disease and smoking status do not predict referral [6, 10], whereas they support that the combination of chronic disease and current smoking may represent a bias in the clinical decision-making [6, 15]. This could indicate that by increasing the awareness of referrals of high-risk patients amongst GPs, timely diagnosis of lung cancer may be enhanced.

The present study found no clear influence of health literacy on diagnostic evaluation. This was surprising, given that individuals with health literacy challenges may find it overwhelming to comply with the demands of the CCPs [29, 30]. GPs often have extensive knowledge about their patients’ life circumstances and prerequisites, including their ability to interact with the healthcare system. Thus, as coordinators in the diagnostic pathway, the GPs can ensure safety-netting and coherence to support the diagnostic evaluation of all patients presenting with relevant symptoms regardless of their health literacy [28, 31].

## Strengths and limitations

The population-based design combining both survey and register data is considered a strength because it allows the analysis of individual factors such as symptoms, health literacy and smoking status. Furthermore, this study enabled the analysis of patients who presented symptoms in general practice but were not referred for diagnostic evaluation or diagnosed with lung cancer, which adds new perspectives to existing evidence.

This study includes some methodological issues. Although the sample size was large and random, the response rate was lower than desired. In addition, we restricted the study population of interest to patients who had presented LCSs in general practice, compromising power and external validity. Both healthcare-seeking behaviour [16, 18] and participation in surveys have been associated with stronger health literacy [32]. Consequently, some degree of selection bias is likely, and the level of health literacy observed in the study population is probably overestimated compared to patients in general practice. This selection bias may cause an underestimation of the effect of health literacy in the present study and a risk of a type II error. Furthermore, selection bias may challenge the generalisability of the findings, and it is possible that referral patterns for patients presenting with LCSs in general practice are less equitable than suggested by our results.

Self-reported smoking status may be compromised by social desirability bias [33]. To mitigate the risk of misclassification, participants were assured of anonymity, and the survey was administered online. However, the absence of data on pack-years represents a limitation of the study, as such information could have provided additional nuances to the analysis. Any chronic disease was assessed self-reported [34], whereas chronic respiratory disease was register based. We hypothesised that respiratory diseases might influence diagnostic imaging to a higher degree than chronic diseases in general. However, findings for both measures of chronic disease were similar.

To comply with the assumption of no unadjusted confounding in the causal mediation model, we adjusted for available confounders under the assumption that the direct and indirect effects are created by unrelated and distinct mechanisms [35]. However, other factors, such as stigmatisation, concern, previous cancer and familial lung cancer, or GP-related factors, such as experience or gut feeling, may also influence the suspicion of cancer and the decision to refer and constitute potential residual confounding. Furthermore, both the stratified results and mediation analyses results should be considered with care due to multiple testing and risk of random findings.

The chosen period for diagnostic imaging could be a moot point. The link between the GP contact reported in the questionnaire and the imaging extracted from the registers is not certain. The broad time span and the overlap between the periods of diagnostic imaging and data collection may introduce a risk of reverse causation. For example, a negative imaging result may influence subsequent symptom reporting. Moreover, as health literacy may have been strengthened during the diagnostic process, the health literacy levels reported by individuals who underwent diagnostic imaging could be inflated. This, in turn, might result in an overestimation of the associations between health literacy strengths and diagnostic imaging. However, since the study did not identify such associations, the impact of this temporal overlap is probably inconsiderable. The broad time span was chosen to ensure the inclusion of all possible relevant imaging, considering that some of the symptoms may have been prolonged, and some patients may have had several GP contacts before referral [36]. Conversely, the exclusion of imaging performed acutely may cause a minor underestimation of diagnostic imaging, as some of the patients may have had severe symptoms, thus prompting the GP to admit the patient immediately. Furthermore, having completed diagnostic imaging in an acute setting may have reduced the likelihood of being referred for further imaging subsequently. However, including patients who completed acute imaging would cause an overestimation of diagnostic imaging, since a high proportion of patients who enter the Danish emergency departments complete a CXR [37]. Other patients may have completed diagnostic imaging prior to the study period. However, the durability for imaging is limited [38]. Therefore, we considered it reasonable to anticipate that relevant diagnostic imaging in relation to the symptoms reported in the survey had been completed within the study period.

### Implications

Initiatives targeting the increased awareness of the risk of overlooking cancer symptoms amongst high-risk patients presenting in general practice may improve timely diagnosis of lung cancer amongst, for instance, patients with chronic diseases. These patients are often monitored in general practice, which provides GPs with an ongoing impression of the disease, persistence and evolvement in symptoms, enabling them to guide the patients and act on changes in symptom presentation.

The present study was unable to determine why patients with a current smoking history do not seem to be more likely to undergo diagnostic imaging when presenting with LCSs in general practice than individuals with no smoking history. Therefore, studies examining consultations in general practice from both patient and GP perspectives may help elucidate decision-making processes.

Healthcare resources are not unlimited; therefore, differentiation and wise use of diagnostic imaging are recommended. Referral of all patients who present with LCSs in general practice for diagnostic imaging is neither cost-effective nor relevant, and few patients will ultimately be diagnosed with cancer [39]. Thus, stratification is preferable in terms of allocating resources to those who need them most and avoiding unnecessary examination with risk of incidental non-specific findings [40].

## Conclusion

Amongst patients who presented symptoms of lung cancer in general practice, diagnostic imaging was completed predominantly by patients who formerly smoked, whereas considerably fewer patients who currently smoked had completed diagnostic imaging. This study could not demonstrate associations between health literacy and diagnostic imaging, and health literacy did not mediate the associations between smoking status and diagnostic imaging. Some interaction was indicated by existing chronic disease lowering the likelihood of patients with a current smoking history completing diagnostic imaging, whereas health literacy strengths seemed to increase the likelihood of completing diagnostic imaging amongst patients who either currently or never smoked.

## Supplementary Material



## Data Availability

The datasets generated and analysed in the current study are not publicly available and cannot be shared due to the data protection regulations of the Danish Data Protection Agency. Access to the data is strictly limited to the researchers who have obtained permission for data processing. This permission was given to the Research Unit of General Practice, Department of Public Health, University of Southern Denmark. Further inquiries can be made to the PI Dorte Jarbøl, email: DJarbol@health.sdu.dk.
