# Supplementary material for: How do health literacy and chronic disease influence the diagnostic evaluation of patients with lung cancer symptoms?
Source: Acta Oncol. 2025 Oct 23;64:44113. doi: 10.2340/1651-226X.2025.44113 (PMC12573666; doi:10.2340/1651-226X.2025.44113)

Supplementary material has been published as submitted. It has not been copyedited, or typeset by Acta Oncologica

Supplementary materials

Supplementary Table S1: Questionnaire and register data

Variable(s)	Question(s)	Answer categories
Symptom experience	*Have you within **the preceding 4 weeks** experienced any of these? (You may tick more than one box)*	Coughing<br>Haemoptysis<br>Dyspnoea<br>Hoarseness<br>Changes to a familiar cough
**Onset**	*When did you experience the symptom for the first time?*	< 1 month ago, 1-3 three months ago, 3-6 months ago and > 6 months ago
**Contact to the general practitioner**	Additional questions asked for each reported symptom:<br>*Have you contacted your general practitioner about any of the following symptoms or discomforts (via telephone, email, video-consultation or clinic visit)*	Yes or no
**Smoking status**	*Do you smoke?*	Yes, every day; Yes, at least once a week Yes, less than once a week; No, I have stopped; No, I have never smoked
**Self-reported chronic disease**	Do you have any chronic disease, long-term effects after injuries, disability or other chronic disorder?	Yes, No, I don't know
	**Health literacy questionnaire***	
**Supported and understood**	*I have at least one healthcare provider who knows me well…*<br>*I have at least one healthcare provider I can discuss…*<br>*I have the healthcare providers I need to help me work…*<br>*I can rely on at least one healthcare provider …*	1=strongly disagree, 2=disagree, 3=agree, 4= strongly agree
**Sufficient information**	*I feel I have good information about health…*<br>*I have enough information to help me deal with…*<br>*I am sure I have all the information I need to manage…*<br>*I have all the information I need to look after my health…*	1=strongly disagree, 2=disagree, 3=agree, 4= strongly agree
**Social support**	*I can get access to several people who understand and…*<br>*When I feel ill, the people around me really understand me...*<br>*If I need help, I have plenty of people I can rely on…*<br>*I have at least one person who can come to medical…*<br>*I have strong support from family and friends…*	1=strongly disagree, 2=disagree, 3=agree, 4= strongly agree
**Actively engage**	*Make sure that healthcare providers understand your…*<br>*Feel able to discuss your health concerns with a…*<br>*Have good discussions about health with doctors…*<br>*Discuss things with healthcare providers until you understand…*<br>*Ask healthcare providers questions to get the…*	1=always difficult, 2=usually difficult, 3=sometimes difficult, 4=usually easy, 5=always easy

Register data

Covariate	Register	Categories
**Sex**	Danish Civil Registration System	Civil Registration System Number Ending with:<br>- even number: female<br>- odd number: male
**Age**	Danish Civil Registration System	Civil Registration System Number<br>- counted at the time of invitation based on birthday.
**Marital status**	Danish Civil Registration System	Single/Living alone<br>Married/Living together
**Educational level**	Danish Education Register	Low: < 10 years<br>Medium: 10-15 years<br>High: >15 years
**Labour market affiliation**	Income Statistics Register	Working<br>Pensioner<br>Out of workforce<br>Disability pension
**Ethnicity**	Danish Civil Registration System	Danish<br>Immigrants or descendants of immigrants
**Chronic Respiratory Disease**	Register of Pharmaceutical Sales	Bronchodilatators: R03AC, R03CC, R03BB<br>Glucocorticosteroids: R03BA,

		Combinations: R03AK, R03AL<br>Leukotrinantagonists: R03DC
**Diagnostic imaging**	Danish National Patient Register	Chest X-ray: UXRC, UXRC00<br>CT of the thorax: UXCC, UXCC0, UXCC75

* The Health Literacy Questionnaire is used under licence, thus only fractions of the questions are allowed to be published.

Table S2: STROBE Statement—Checklist of items that should be included in reports of ***cross-sectional studies***

	Item No	Recommendation
**Title and abstract**	1	(*a*) Indicate the study's design with a commonly used term in the title or the abstract (2)
		(*b*) Provide in the abstract an informative and balanced summary of what was done and what was found (2)
**Introduction**		
Background/rationale	2	Explain the scientific background and rationale for the investigation being reported (3)
Objectives	3	State specific objectives, including any prespecified hypotheses (4)
**Methods**		
Study design	4	Present key elements of study design early in the paper (4)
Setting	5	Describe the setting, locations, and relevant dates, including periods of recruitment, exposure, follow-up, and data collection (4,5)
Participants	6	(*a*) Give the eligibility criteria, and the sources and methods of selection of participants (6)
Variables	7	Clearly define all outcomes, exposures, predictors, potential confounders, and effect modifiers. Give diagnostic criteria, if applicable (6, 7)
Data sources/ measurement	8*	For each variable of interest, give sources of data and details of methods of assessment (measurement). Describe comparability of assessment methods if there is more than one group (4-7)
Bias	9	Describe any efforts to address potential sources of bias (4, 10-11)
Study size	10	Explain how the study size was arrived at (7, 8)
Quantitative variables	11	Explain how quantitative variables were handled in the analyses. If applicable, describe which groupings were chosen and why (6,7)
Statistical methods	12	(*a*) Describe all statistical methods, including those used to control for confounding (7)
		(*b*) Describe any methods used to examine subgroups and interactions (7)
		(*c*) Explain how missing data were addressed (6, 7)
		(*d*) If applicable, describe analytical methods taking account of sampling strategy
		(*e*) Describe any sensitivity analyses
**Results**		
Participants	13*	(a) Report numbers of individuals at each stage of study—eg numbers potentially eligible, examined for eligibility, confirmed eligible, included in the study, completing follow-up, and analysed (8)
		(b) Give reasons for non-participation at each stage (8)

		(c) Consider use of a flow diagram (Figure 2)
Descriptive data	14*	(a) Give characteristics of study participants (eg demographic, clinical, social) and information on exposures and potential confounders (8)
		(b) Indicate number of participants with missing data for each variable of interest (8)
Outcome data	15*	Report numbers of outcome events or summary measures (8)
Main results	16	(*a*) Give unadjusted estimates and, if applicable, confounder-adjusted estimates and their precision (eg, 95% confidence interval). Make clear which confounders were adjusted for and why they were included (8-9)
		(*b*) Report category boundaries when continuous variables were categorized
		(*c*) If relevant, consider translating estimates of relative risk into absolute risk for a meaningful time period
Other analyses	17	Report other analyses done—eg analyses of subgroups and interactions, and sensitivity analyses (9)
**Discussion**		
Key results	18	Summarise key results with reference to study objectives (9)
Limitations	19	Discuss limitations of the study, taking into account sources of potential bias or imprecision. Discuss both direction and magnitude of any potential bias (10-12)
Interpretation	20	Give a cautious overall interpretation of results considering objectives, limitations, multiplicity of analyses, results from similar studies, and other relevant evidence (10-12
Generalisability	21	Discuss the generalisability (external validity) of the study results (10)
**Other information**		
Funding	22	Give the source of funding and the role of the funders for the present study and, if applicable, for the original study on which the present article is based (14)

Figure S1: Directed Acyclic Graph for the causal mediation models

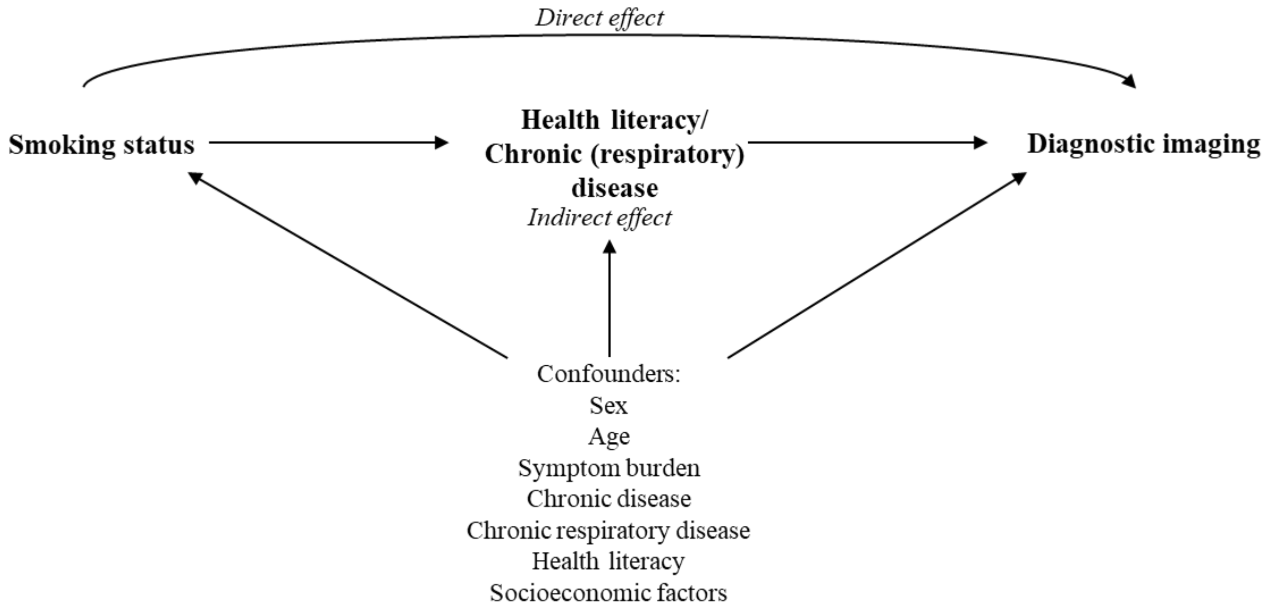

Supplement: Supplementary file 1 [file AO-60-44113-s1.pdf]
